# Supplementary material for: One single drug-coated balloon for all shapes/diameters? Neointimal proliferation inhibition in porcine peripheral arteries
Source: PLoS One. 2023 Jan 27;18(1):e0280206. doi: 10.1371/journal.pone.0280206 (PMC9882906; doi:10.1371/journal.pone.0280206)
Supplement: S1 Table — (DOCX) [file pone.0280206.s001.docx]

**Supporting information**

**S1 Table.**

|  | **Hyper-compliant balloon** | | | | | **Standard PTA balloon** | | | **p-value** | **Hyper-compliant balloon** | | | **Standard PTA balloon** | **p-value** |  |
| --- | --- | --- | --- | --- | --- | --- | --- | --- | --- | --- | --- | --- | --- | --- | --- |
|  | **Uncoated (HCB)** | **Coated (HCDCB)** | | | **Coated (DCB)** | | |  | | **Uncoated (HCB)** | **Coated (HCDCB)** | **Coated (DCB)** | |  | |
|  | **Femoral proximal** | | | | | | | | | **Femoral distal** | | | | | |
| **n (analyzed vessels)** | - | - | | | - | | |  | | 6^*♦^ | 8 | 7^*^ | |  | |
| **Lumen area [mm^2^]** | Measurement not possible due to methological limitations | | | | | | | | | 7.66±1.69 | 9.29±1.45 | 8.74±1.86 | | 0.218 | |
| **Stent area [mm^2^]** |  |  |  |  |  |  |  |  |  | 10.6±1.1 | 11.0±1.5 | 10.8±1.4 | | 0.869 | |
| **Neointimal area [mm^2^]** |  |  |  |  |  |  |  |  |  | 2.93±1.55 | 1.70±0.64 | 2.03±0.96 | | 0.121 | |
|  | **Internal iliac proximal** | | | | | | | | | **Internal iliac distal** | | | | | |
| **n (analyzed vessels)** | 8 | | 8 | 8 | | |  | | | 7^*^ | 8 | 7^**^ | |  | |
| **Lumen area [mm^2^]** | 5.86±1.31 | | 8.58±1.70^a^ | 7.73±1.57 | | | 0.006 | | | 3.05±0.71 | 3.79±0.65 | 4.08±0.88^a^ | | 0.049 | |
| **Stent area [mm^2^]** | 8.58±1.46 | | 9.93±1.65 | 9.29±1.84 | | | 0.285 | | | 5.66±0.87 | 5.87±1.08 | 6.37±1.09 | | 0.426 | |
| **Neointimal area [mm^2^]** | 2.72±1.92 | | 1.36±0.27 | 1.56±0.52 | | | 0.060 | | | 2.61±0.87 | 2.08±0.79 | 2.29±0.90 | | 0.487 | |

^*^ One vessel segment not treated by a balloon; ^**^ one vessel segment not evaluated due to outflow obstruction, ^♦^ imaging catheter not advanceable into one vessel segment.

Data presented as mean ± SD. p-values were calculated with one-way ANOVA with post-hoc analysis (Tukey). ^a^ Significant difference to HCB.
