# Supplementary material for: One single drug-coated balloon for all shapes/diameters? Neointimal proliferation inhibition in porcine peripheral arteries
Source: PLoS One. 2023 Jan 27;18(1):e0280206. doi: 10.1371/journal.pone.0280206 (PMC9882906; doi:10.1371/journal.pone.0280206)
Supplement: S2 Table — (DOCX) [file pone.0280206.s002.docx]

**Supporting information**

**S2 Table.**

|  | **Hyper-compliant balloon** | | **Standard PTA balloon** | **p-value** | **Hyper-compliant balloon** | | | **Standard PTA balloon** | **p-value** |  |
| --- | --- | --- | --- | --- | --- | --- | --- | --- | --- | --- |
|  | **Uncoated (HCB)** | **Coated (HCDCB)** | **Coated (DCB)** |  | **Uncoated (HCB)** | **Coated (HCDCB)** | **Coated (DCB)** | |  | |
|  | **Femoral proximal** | | | | **Femoral distal** | | | | | |
| **n (analyzed vessels)** | 8 | 8 | 8 |  | 7^*^ | 8 | 7^*^ | |  | |
| **Vessel diameter [mm]** | 4.68±0.28 | 4.51±0.31 | 4.76±0.16 | 0.159 | 4.05±0.22 | 4.05±0.29 | 4.09±0.17 | | 0.941 | |
| **Lumen diameter [mm]** | 3.37±0.59 | 3.21±0.34 | 3.58±0.53 | 0.346 | 2.83±0.47 | 3.07±0.32 | 3.01±0.49 | | 0.522 | |
| **Lumen area [mm^2^]** | 9.39±3.24 | 8.43±1.74 | 10.4±2.9 | 0.370 | 6.34±2.11 | 7.43±1.62 | 7.34±2.07 | | 0.505 | |
| **Neointimal area [mm^2^]** | 7.67±1.91 | 7.45±1.39 | 7.36±2.04 | 0.937 | 6.27±1.38 | 5.29±1.31 | 5.42±1.30 | | 0.336 | |
|  | **Internal iliac proximal** | | | | **Internal iliac distal** | | | | | |
| **n (analyzed vessels)** | 8 | 8 | 8 |  | 7^*^ | 8 | 7^**^ | |  | |
| **Vessel diameter [mm]** | 3.49±0.34 | 3.51±0.13 | 3.54±0.37 | 0.948 | 2.97±0.10 | 2.90±0.29 | 3.05±0.25 | | 0.513 | |
| **Lumen diameter [mm]** | 2.17±0.20 | 2.62±0.26^a^ | 2.62±0.35^a^ | 0.004 | 1.95±0.12 | 1.99±0.38 | 2.12±0.37 | | 0.587 | |
| **Lumen area [mm^2^]** | 3.74±0.72 | 5.43±0.88^a^ | 5.56±1.69^a^ | 0.009 | 3.00±0.42 | 3.04±1.30 | 3.51±1.24 | | 0.619 | |
| **Neointimal area [mm^2^]** | 5.94±2.09 | 4.29±0.71 | 4.25±0.80 | 0.032 | 3.95±0.68 | 3.33±0.54 | 3.65±0.73 | | 0.214 | |

^*^ One vessel segment not treated by a balloon; ^**^ one vessel segment not evaluated due to outflow obstruction.

Data presented as mean ± SD. p-values were calculated with one-way ANOVA with post-hoc analysis (Tukey). ^a^ Significant difference to HCB.
