# Supplementary material for: One single drug-coated balloon for all shapes/diameters? Neointimal proliferation inhibition in porcine peripheral arteries
Source: PLoS One. 2023 Jan 27;18(1):e0280206. doi: 10.1371/journal.pone.0280206 (PMC9882906; doi:10.1371/journal.pone.0280206)
Supplement: Attachment — Submitted filename: Response to reviewers 241122.docx [file pone.0280206.s004.docx]

**Rebuttal letter to manuscript PONE-D-22-23006**

I) Editor Comments

Can the authors please provide some discussion with regards to future plans and methodology for human testing? Please refer to Reviewer 1 comments.

Our response

Possible clinical scenarios are discussed in the Conclusions section.

II) Reviewer #1

… The balloon lengths used in the animal study were 10-15 cm, but in humans, one would hope that 30-40 cm balloons would be feasible in order to treat the entire popliteal and superficial femoral arterial section in one go. It would be good for the authors to discuss in their conclusions and the plan for in human testing.

Our response

We have addressed the reviewer’s #1 view regarding the balloon lengths used in our animal study by stating that the use of balloons of shorter lengths (i.e., 10-15 cm) in our animal study is related to the size and anatomy of the experimental animals (see section ‘Limitations’, lines 427-431).

III) Reviewer #2

… However application of such device in humans is still under investigation especially in patients with long lesions which are either stenotic of occluded with high calcific burden.

a) Do the authors believe that HCDCB are effective in calcified lesions where drug delivery across the arterial wall is low?

Our response

We have added a statement concerning calcium being a barrier to drug transfer and vessel wall distribution, and a corresponding citation (Fanelli et al., 2014 [15]) (see section ‘Conclusions’, lines 448-450).

b) Do the authors believe that HCDCB at low pressures can fully accommodate to different diameter lesion in humans?

Our response

We have observed in our several animal experiments that at low inflation pressure long hyper-compliant balloons (HCDCB) well adapt to the vessel structure regardless of vessel diameters, also by forming protrusions at vessel branches. A statement addressing this issue is read in the section ‘Conclusions’.

c) Pg3 line 63 Please correct vessles to vessels

Our response

This correction has been made.
